# Microbiota-derived short-chain fatty acids do not interfere with SARS-CoV-2 infection of human colonic samples

**DOI:** 10.1080/19490976.2021.1874740

**Published:** 2021-02-08

**Authors:** Lívia Bitencourt Pascoal, Patrícia Brito Rodrigues, Lívia Moreira Genaro, Arilson Bernardo dos Santos Pereira Gomes, Daniel Augusto Toledo-Teixeira, Pierina Lorencini Parise, Karina Bispo-Dos-Santos, Camila Lopes Simeoni, Paula Veri Guimarães, Lucas Ildefonso Buscaratti, João Gabriel De Angeli Elston, Henrique Marques-Souza, Daniel Martins-de-Souza, Maria De Lourdes Setsuko Ayrizono, Lício Augusto Velloso, José Luiz Proenca-Modena, Pedro Manoel Mendes Moraes-Vieira, Marcelo Alves Silva Mori, Alessandro Santos Farias, Marco Aurélio Ramirez Vinolo, Raquel Franco Leal

**Affiliations:** a Laboratory of Immunoinflammation, Department of Genetics, Microbiology and Immunology, Institute of Biology, University of Campinas (UNICAMP), Campinas, Brazil; b Inflammatory Bowel Disease Research Laboratory, Colorectal Surgery Unit, Department of Surgery, School of Medical Sciences, University of Campinas (UNICAMP), Campinas, Brazil; c Laboratory of Emerging Viruses, Department of Genetics, Microbiology and Immunology, Institute of Biology, University of Campinas (UNICAMP), Campinas, Brazil; d Brazilian Laboratory on Silencing Technologies (Blast), Department of Biochemistry and Tissue Biology, Institute of Biology, University of Campinas (UNICAMP), Campinas, Brazil; e Experimental Medicine Research Cluster (EMRC), University of Campinas (UNICAMP), Campinas, Brazil; f Laboratory of Neuroproteomics, Department of Biochemistry and Tissue Biology, Institute of Biology, University of Campinas (UNICAMP), Campinas, Brazil; g Laboratory of Cell Signaling, School of Medical Sciences,University of Campinas (UNICAMP), Campinas, Brazil; h Obesity and Comorbidities Research Center (OCRC), University of Campinas (UNICAMP), Campinas, Brazil; i Laboratory of Immunometabolism, Department of Genetics, Microbiology and Immunology, Institute of Biology, University of Campinas (UNICAMP), Campinas, Brazil; j Laboratory of Aging Biology, Department of Biochemistry and Tissue Biology, Institute of Biology, University of Campinas (UNICAMP), Campinas, Brazil; k Autoimmune Research Laboratory, Department of Genetics, Microbiology and Immunology, Institute of Biology, University of Campinas (UNICAMP), Campinas, Brazil

**Keywords:** SARS-CoV-2, COVID-19, microbiota, short-chain fatty acids, human colonic samples

## Abstract

Microbiota-derived molecules called short-chain fatty acids (SCFAs) play a key role in the maintenance of the intestinal barrier and regulation of immune response during infectious conditions. Recent reports indicate that SARS-CoV-2 infection changes microbiota and SCFAs production. However, the relevance of this effect is unknown. In this study, we used human intestinal biopsies and intestinal epithelial cells to investigate the impact of SCFAs in the infection by SARS-CoV-2. SCFAs did not change the entry or replication of SARS-CoV-2 in intestinal cells. These metabolites had no effect on intestinal cells’ permeability and presented only minor effects on the production of anti-viral and inflammatory mediators. Together our findings indicate that the changes in microbiota composition of patients with COVID-19 and, particularly, of SCFAs do not interfere with the SARS-CoV-2 infection in the intestine.

## Introduction

COVID-19 is a pandemic disease caused by severe acute respiratory syndrome coronavirus 2 (SARS-Cov-2), characterized as respiratory disorder with clinical changes ranging from no symptoms to severe pneumonia and death.^1^^,2^ After an incubation period, most patients with COVID-19 develop mild-to-moderate disease with typical symptoms including fever, chills, fatigue, dry cough, sore throat, sputum production, shortness of breath and headache.^2,3^ In addition, recent studies showed that 17.6% of patients with COVID-19 present gastrointestinal symptoms that occurred more frequently in severe patients.^3,4^ Interestingly, the presence of SARS-CoV-2 in fecal samples was associated with changes in gut microbiota composition.^5^ Numerous experimental and clinical observations suggested that the gut microbiota plays a key role in the pathogenesis of sepsis and acute respiratory distress syndrome suggesting that SARS-CoV2 might also have an impact on the gut microbiota and vice-versa.^5–7^

Loss of gut bacteria diversity leading to dysbiosis is associated with the development of many diseases.^5–7^ This also seems to be the case for SARS-CoV-2 infection. A recent study reported an increase of opportunistic bacteria such as *Collinsella aerofaciens, Collinsella tanakaei, Streptococcus infantis* and *Morganella morganii* and a reduction of *Parabacteroides merdae, Bacteroides stercoris, Alistipes onderdonkii* and *Lachnospiraceae bacterium*1_1_57FAA) in patients with high SARS-CoV-2 infectivity signature compared to patients with low or no SARS-CoV-2 infectivity.^5^ Functionally, this change in microbiota composition was associated with a reduction in short-chain fatty acids (SCFAs) production and increased synthesis of nucleotide and amino acids and carbohydrate metabolism. Another study pointed out to a reduction in bacterial groups (e.g., *Faecalibacterium, Fusicatenibacter* and *Eubacterium hallii*) involved in the production of the SCFA butyrate in fecal samples of COVID-19 patients compared to healthy controls.^6^ Thus, there is evidence that the presence and/or infection of SARS-CoV-2 in the gut is associated with changes in microbiota including reduction in SCFAs-producing bacteria. However, no study addressed whether this effect on SCFAs is relevant for the infection.

Butyrate and other SCFAs are key molecules mediating host–microbiota interaction. Previous studies reported the ability of these molecules to regulate the production of antimicrobial peptides and mucus, intestinal permeability and mucosal immune system activation.^8^ The gastrointestinal tract deserves special attention, in particular the potential role of the gut microbiota in the development and management of this disease. Therefore, we hypothesized that a reduction in SCFAs’ production would affect SARS-CoV-2 entry and response of intestinal cells.

## Results

### Treatment with SCFAs does not affect the entry of SARS-CoV-2 or the response of the intestinal tissue to infection

We used human colon biopsies obtained from healthy individuals for investigating the interaction between SARS-CoV-2, microbiota-derived metabolites and intestinal cells (Table 1). Colonic biopsies are an attractive model for this type of study because they allow us to analyze the impact of infection in a well-preserved tissue architecture that includes the colonic epithelium and its lamina propria. To reduce the effect of technical and biological aspects associated with the tissue, we used samples obtained from the same individual that were treated and infected *ex vivo* in the same experimental conditions. Biopsies were maintained in culture for up to 7 h and presented normal histological features after this period of incubation.

**Table 1.** Demographic and clinical characteristics of the patients included in the study

Number of participants	11
Gender (M/F)	2/9
Age (y)	43 [19–65]
Body mass index (kg/m^2^)	25.03 [21.73–26.56]
Smoking	-
Hemoglobin (g/dL)	12.90 [6.6–14.6]
Hematocrit (%)	38.85 [22.8–44.4]
Platelet (×10^3^)	269.50 [158–395]
Albumin (g/dL)	4.20 [3.4–4.4]

**Figure 1.**
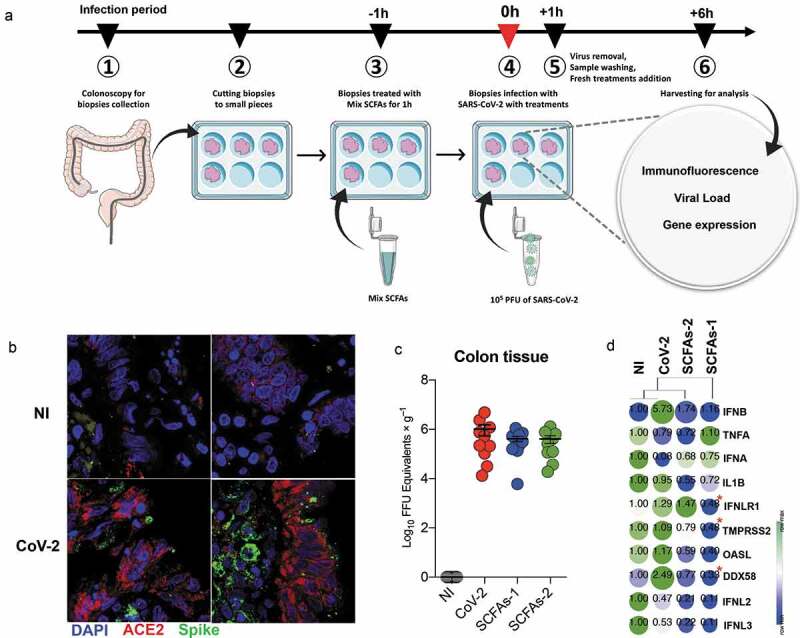
Effect of the SCFAs on the SARS-Cov-2 infection and cytokine response by the intestinal mucosa. (a) Schematic illustration of experimental design. (b) Biopsies infected or not with SARS-CoV-2 and stained for ACE2 (red) and spike (green). Nucleus of cells are identified by DAPI (blue). (c) The viral load was measured in colon biopsies infected with SARS-CoV-2 and incubated in the presence or absence of SCFAs (SCFAs-1 [acetate 16 mM, propionate 4 mM and butyrate 2 mM] and SCFAs-2 [acetate 1.6 mM, propionate 0.4 mM and butyrate 0.2 mM]). Noninfected (NI) biopsies were used as negative controls of the experiments. Results are presented as mean ± SEM (*n* = 10 individuals/group). (d) Gene expression in colon biopsies infected or not with SARS-CoV-2 and incubated in the presence or absence of SCFAs. The expression of genes related to the entry of SARS-CoV-2 (TMPRSS2), inflammation (IL1b and TNF), virus recognition (DDX58) and response (type III interferon and its receptor – IFNL2, IFNL3 and INFR1, respectively – type I interferon – IFN beta and IFN alpha), and IFN target genes related to virus elimination (OASL) were analyzed by RTq-PCR. Results were normalized by the NI condition and are presented as mean (*n* = 9–12 individuals/group). *p < .05 compared to SARS-CoV-2

Immunofluorescence staining revealed that the cells from colonic biopsies expressed the SARS-CoV-2 receptor, the angiotensin-converting enzyme-2 (ACE2, in red), and were efficiently infected by the virus, as shown by the spike staining (green) (Figure 1b). This later finding was confirmed by the measurement of virus load (Figure 1c). Colonic biopsies treated with different concentrations of SCFAs presented the same viral load as the control condition, indicating that these metabolites do not interfere with virus entrance in cells (Figure 1c).

Previous studies in human intestinal organoids infected with SARS-CoV-2 reported increased production of type-I and III interferon (IFN), cytokines that are relevant for the antiviral response.^9–11)^ Therefore, we evaluated the expression of these cytokines and of inflammatory-related genes in the colonic biopsies. We observed an increase of DDX58, a gene which encodes the viral receptor RIG-I (retinoic acid-inducible gene I), and of IFN beta, in infected biopsies compared with noninfected (Figure 1d). When compared to the infected biopsies, we verified a significant reduction of DDX58 and the type III IFN receptor, IFNLR1, in biopsies treated with SCFAs at the higher concentration (SCFAs 1, Figure 1d). We also observed a reduction in the expression of the serine protease TMPRSS2, a protein that is important for SARS-CoV-2 entry into target cells.^12^ The expression of other antiviral and inflammatory genes was not modulated by the SCFAs (Figure 1d).

**Figure 2.**
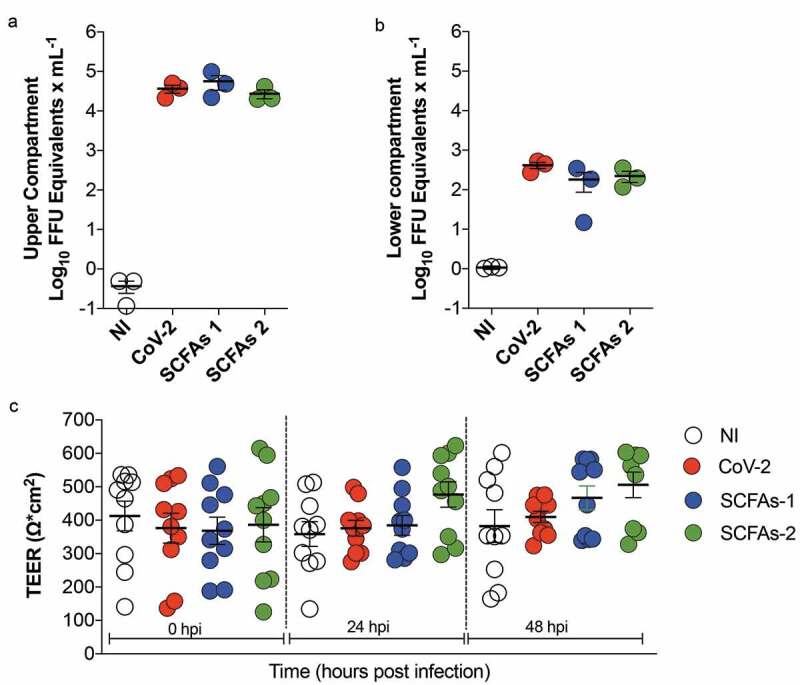
Effect of the SCFAs on SARS-CoV-2 infection and transepithelial resistance of Caco-2. Caco-2 polarized cells were treated with SCFAs (SCFAs-1 [acetate 8 mM, propionate 2 mM and butyrate 1 mM] and SCFAs-2 [acetate 4 mM, propionate 1 mM and butyrate 0.5 mM]) and infected with SARS-CoV-2. Noninfected (NI) cells were used as negative controls of the experiments (a, b) Viral load was measured by RT-PCR in the upper and lower compartment of Caco-2 monolayer (*n* = 3) at the end of the experiment (48 h after incubation) (*n* = 3). (c) Transepithelial resistance of Caco-2 was measured at 24 and 48 h after infection (*n* = 10). Symbols represent individual samples. Results are presented as mean ± SEM. Data presented in (a) and (b) are representative of two independent experiments

We next investigated the effect of SARS-CoV-2 and SCFAs on isolated intestinal epithelial cells (Caco-2). For that, we used Caco-2 cells cultivated for 2–3 weeks in transwell inserts. Under this condition, cells differentiate and form a polarized monolayer, whose permeability/integrity can be measured by the transepithelial electrical resistance (TEER). In these experiments, we did not observe any effect of SARS-CoV-2 infection or the SCFAs on transepithelial resistance of Caco-2 monolayers (Figure 2c). We also measured the amount of virus released in both apical and basolateral surfaces of infected cells and did not find any effect of SCFAs in these parameters (Figure 2a and b). Taken together, our results indicate that SCFAs do not affect the entry, replication or the intestinal cells’ response to SARS-CoV-2 infection.

## Discussion

Patients with severe forms of COVID-19 frequently manifest gastrointestinal symptoms such as diarrhea, vomiting and abdominal pain.^13–15^ Moreover, gut microbiota composition is altered in most COVID-19 patients, and it is neither known if this could worsen the clinical course of the disease, nor if the microbiota modulation could help to restore a balanced immune response against this viral infection.^16,17^ Many studies have already been carried out looking for the effects of SCFAs in the treatment of infections, including viral airway infections. The consumption of a high-fiber diet or oral supplementation with acetate protected mice from infection by the respiratory syncytial virus (RSV) through GPR43 activation and IFN beta production in the lung epithelial cells.^18^ Butyrate, as well as treatment with a high-fiber diet, was shown to protect mice from influenza infection by modulating their immune response.^19^ Treatment of vascular endothelial cells with SCFAs decreased the expression of VCAM-1 and ICAM-1, resulting in reduced adhesion of infected monocytes and virus transfer to the endothelium.^20^ Acetate treatment during Influenza infection was effective in reducing secondary bacterial pulmonary infections.^21^ Based on this evidence gathered before the SARS-CoV-2 pandemic, many researchers indicated that the reestablishment of SCFAs endogenous production could be useful for the prevention and treatment of COVID-19.^16,17,22^ However, it is worth mentioning that detrimental effects of SCFAs on virus infections have also been reported. A recent study demonstrated that butyrate increases cellular infection by H1N1 influenza A virus, reovirus and human immunodeficiency virus 1 (HIV-1). This effect was associated with suppression of specific antiviral interferon-stimulated genes.^23^ Another study reported an exacerbation of arthropathy-induced by Chikungunya virus in mice after treatment with high-fiber diet or butyrate.^24^

Using colon biopsies from patients who were diagnosed with SARS-CoV-2 a few days after colonoscopy, it was possible to observe that intestinal cells are infected with the new coronavirus.^25^ Other studies involving human intestinal organoid experiments confirmed the mechanism of viral entry into intestinal cells, as well as the molecular expression pattern associated with the viral invasion in a context of intestinal inflammation (patients with inflammatory bowel diseases).^26,27^ Differences in the expression of molecules related to viral entry depended on the analyzed intestinal segment, ileum or colon.^27^

In the present study, the treatment with a mixture of acetate, propionate, and butyrate did not alter the viral load of intestinal biopsies or intestinal epithelial cells. These findings do not exclude the possibility that the SCFAs have a significant effect on SARS-CoV-2 infection. The antiviral effects promoted by the microbiota and its metabolites may depend on the interactions with different cell types and further studies are needed to understand these mechanisms during SARS-CoV-2 infection.

One of the characteristics of COVID-19 disease is the exacerbated inflammatory response that occurs in a second phase of the disease. Thus, one of the main investigations that has been carried out around the world is to dissect how the infection occurs in each tissue and systems and how that tissue reacts to the presence of this infection, especially in patients who already have an inflammatory condition, such as obesity.^23,28,29^ A study with intestinal and pulmonary epithelial cell lines showed that SARS-CoV-2 infection alters the expression of inflammatory cytokines and anti-viral molecules such as IFNα and IFNβ in lung cells. Their findings suggested the pre-activation of IFN-I signaling pathway as a potential therapeutic and prophylactic management for COVID-19^30^. In our study, the treatment with SCFAs reduced the transcript levels of genes important for the detection of viral molecules, control of viral entry and replication, such as RIG1, TMPRSS2, and the IFNλ receptor. However, the viral load of SCFAs-treated samples did not differ from the nontreated infected biopsies indicating that these effects are not sufficient or may be counteracted by other effects of SCFAs on these cells.

Some limitations of our study should be noted, such as the small sample size and the lack of intestinal biopsies from patients with COVID-19. However, the use of human samples, even of noninfected patients, provides a relevant contribution to establish a potential role of SCFAs in this pandemic disease.

Our results need to be validated *in vivo*, but indicate that changes in microbiota composition of patients with COVID19^5,6^ and, particularly, of SCFAs do not interfere with the SARS-CoV-2 infection in the intestine. It is worth mentioning that SCFAs can also have systemic effects, which may be relevant for SARS-CoV-2 infection in different contexts.^31^

## Materials and methods

### Patient and sample selection

Left colon mucosa biopsies were collected from patients who underwent colonoscopy examination for diagnostic purposes and who presented no endoscopic abnormalities. All subjects were recruited at the Gastrocenter’s Colonoscopy Unit of the Clinic Hospital from University of Campinas (Unicamp) and included in this study after having signed a written informed consent form. Table 1 shows the clinical and demographic characteristics of the 12 patients without comorbidities who participated in the study.

## Virus

Low passage of strain HIAE-02-SARS-CoV-2/SP02/human/2020/bra (GenBank MT126808) kindly donated by Prof. Dr. Edison Durigon (ICB-USP) was propagated in Vero cells (ATCC CCL81) for using in all experiments at the biosafety level 3 area of the Laboratory of Emerging Viruses (IB-Unicamp).

## Culture of intestinal biopsies

Immediately after the mucosa biopsies were collected during the colonoscopy examination, they were washed and included in culture. Culture of intestinal biopsy specimens was performed in RPMI-1640 medium (Sigma-Aldrich, Germany) without L-glutamine and supplemented with 10% fetal calf serum and antibiotic/antimycotic mixture (Gibco Invitrogen). The samples were divided in four different conditions: noninfected (medium only), infected with SARS-CoV-2 and treated with short-chain fatty acids at two different concentrations (SCFAs-1 [acetate 16 mM, propionate 4 mM and butyrate 2 mM] or SCFAs-2 [acetate 1.6 mM, propionate 0.4 mM and butyrate 0.2 mM]), and infected with SARS-CoV-2. The ratio of SCFAs (acetate, propionate and butyrate) used in the study was similar to what was described in a previous study that measured these metabolites in fecal samples.^32^ The concentrations of SCFAs were chosen based on experiments performed with Caco-2 cells in which we found that incubation for 24 h with SCFAs did not affect their viability.

All infections were performed with 10^5^ PFU of SARS-CoV-2 for 1 h at room temperature (20–25°C) with continuous and gentle agitation. After viral adsorption, samples were washed three times with PBS 1x (0.15 M) and incubated for 6 h at 37°C and 5% of CO_2_ atmosphere with related media conditions. The experimental design of culture and different treatments are illustrated in Figure 1a.

## Cell culture

Human colon cancer cells (Caco-2) seeded 2 × 10^4^ cells per inserts into transwell 24-well plate (0.4 µm polycarbonate membrane with 0.33 cm^2^ area, Costar). Cells were maintained in Dulbecco’s modified Eagle medium (Gibco) supplemented with 20% fetal bovine serum (FBS) and 1% Penicillin-Streptomycin at 37°C and 5% CO_2_ atmosphere for up to 21 d with changes of medium every 2 d. The medium volume in the up chamber was 0.2 mL and in the basal chamber was 0.5 mL. After 21 d of differentiation, cells were pretreated for 1 h with SCFAs (SCFAs-1 [8 mM de acetate, 2 mM de propionate and 1 mM de butyrate] and SCFAs-2 [4 mM de acetate, 1 mM propionate and 0.5 mM butyrate]) or medium alone. Cells were then infected with MOI of 1 at room temperature for 1 h with continuous and gentle agitation. Before viral adsorption, SARS-CoV-2 inoculum was removed, cells were washed three times with PBS 1x and then maintained with related media. Transepithelial resistance was measured immediately after infection (time 0), 24- and 48-h post-infection, as previously described.^33,34^

## RNA extraction and quantification

Total RNA was extracted from colonic mucosa samples and culture supernatants using RNeasy Mini Kit (Qiagen, USA) according to the manufacturer’s instructions. For qPCR analysis, RNA purity and concentration were determined by UV spectrophotometry at 260 nm using the BioTek Eon Microplate Spectrophotometer and Gen5 v 2.0 software.

## Viral load quantification

Viral RNA was detected and quantified by Charité protocol of one-step RT-Qpcr3^34^ using 3 μL of TaqMan Fast Virus 1-Step Master Mix (Applied Biosystems), 800 nM of primers (Forward: 5-ACA GGT ACG TTA ATA GTT AAT AGC GT-3; Reverse: 5-ATA TTG CAG CAG TAC GCA TAC GCA CAC A-3), 400 nM of probe (Probe: 5–6FAM-ACA CTA GCC ATC CTT ACT GCG CTT CG-QSY-3) and 6 μL of RNA samples. The cycling method for running was: 1 cycle of 50°C for 10 min, 1 cycle of 95°C for 2 min, followed by 45 cycles of 95°C for 5 s and 60°C for 30 s in the QuantStudio3 System (Applied Biosystems). Negative and positive control samples were included in every run.

## Gene expression by RT-qPCR

For cDNA synthesis, the High Capacity cDNA Reverse Transcription Kit (Applied Biosystems, Foster City, CA, USA) was used according to the manufacturer’s instructions. qPCR reactions were performed using the TaqMan™ system (Applied Biosystems) for the following primers: IL1b (Hs_01555410_m1), TNFA (Hs_00174128_m1), GAPDH (4326317E), IFNL2 (Hs00820125) and IFNL3 (Hs04193048), and using Power SYBR Green PCR Master Mix (Applied Biosystems) for the following primers: DDX58 (F: CACCTCAGTTGCTGATGAAGGC and R: GTCAGAAGGAAGCACTTGCTACC), OASL (F: GTGCCTGAAACAGGACTGTTGC and R: CCTCTGCTCCACTGTCAAGTGG), IFNA (F: GTACTGCAGAATCTCTCCTTT CTCCTG and R: GTGTCTAGATCTGACAACCTCCCAGGCACA), IFNB1 (F: TTGTGCTTCTCCACTACAGC and R: CTGTAAGTCTGTTAATGAAG), TMPRSS2 (F: CAAGTGCTCCAACTCTGGGAT and R: AACACACCGATTCTCGTCCTC), IFNLR1 (F: ACCTATTTTGTGGCCTATCAGAGCT and R: CGGCTCCACTTCAAAAAGGTAAT). qPCR was performed with the StepOnePlus System (Applied Biosystems) using the TaqMan Fast Advanced master mix (Life Technologies) or Power SYBR Green PCR Master Mix (Applied Biosystems). All measurements were normalized by the expression of the GAPDH gene using the 2(-Delta Delta C(T)) method.

## Immunofluorescence

Colon biopsies were fixed in paraformaldehyde 4% for 24 h and then embedded in paraffin. Five-micrometer-thick sections were prepared for immunoflurescent detection of ACE2 and viral spike protein. Samples were deparaffinized by two 10 min-incubation with Xylol, followed by an incubation with xylol:ethanol (1:1) solution for 10 min, followed by incubations with different concentrations of Ethanol solution (ethanol 100%, ethanol 95%, ethanol 85% and ethanol 70%, respectively, all diluted in DEPC), for 5 min each, and finalizing with water DEPC for 5 min and two times PBS 1x pH 7.4 for 5 min. To avoid autofluorescence, the tissues were treated with 2% H_2_O_2_ methanol for 30 min, washed with PBST, and treated with glycine 0.1 M in PBST for 10 min at room temperature. The samples were then washed and treated with 1% bovine serum albumin (BSA) solution in PBST for 30 min, to block nonspecific epitopes. Tissues were incubated with SARS-CoV-2 Spike S1 Antibody (HC2001) (GenScript – A02038) and ACE2 Antibody (Rheabiotec – IM-0060, both diluted 1:100 in BSA 1% solution in PBST, and incubated overnight at 4°C in a humid box. The slides were then washed and incubated with anti-human IgG Alexa 488 (ThermoFisher – A11013) and anti-rabbit IgG Alexa Fluor 594 (ThermoFisher – A21207), both diluted 1:500 in BSA 1% solution in PBST for 2 h at room temperature in a humid box, protected from the light. The samples were washed again, incubated DAPI (Santa Cruz Biotechnology – SC-3598) diluted 1:1000 in BSA 1% solution in PBST for 5 min at room temperature protected from the light, and mounted in an aqueous mounting solution for confocal imaging. 

Microscopy images were acquired with a Zeiss LSM880 with Airyscan on an Axio Observer 7 inverted microscope (Carl Zeiss AG, Germany) with a C Plan Apochromat 63x/1.4 Oil DIC objective, 4x optical zoom. Prior to image analysis, raw.czi files were automatically processed into deconvoluted Airyscan images using Zen Black 2.3 software. For DAPI were acquired conventional confocal image using 405-nm laser line for excitation and pinhole set to 1 AU.

## Statistical analysis

Analyses were performed using GraphPad software 8.0 (San Diego, CA, USA). Results are presented as mean ± standard error mean (SEM) and “n” represents the number of samples, as indicated in the corresponding figure legend. Differences were considered significant for *p* < .05. Results were compared by non-parametric Mann–Whitney test.

## Data Availability

All relevant data supporting the findings of this study are available within this report.
